# Effects of whole-body electromyostimulation combined with individualized nutritional support on body composition in patients with advanced cancer: a controlled pilot trial

**DOI:** 10.1186/s12885-018-4790-y

**Published:** 2018-09-12

**Authors:** Kristin Schink, Hans J. Herrmann, Raphaela Schwappacher, Julia Meyer, Till Orlemann, Elisabeth Waldmann, Bernd Wullich, Andreas Kahlmeyer, Rainer Fietkau, Dorota Lubgan, Matthias W. Beckmann, Carolin Hack, Wolfgang Kemmler, Jürgen Siebler, Markus F. Neurath, Yurdagül Zopf

**Affiliations:** 1 Department of Medicine 1 – Gastroenterology, Pneumology and Endocrinology, Friedrich-Alexander-Universität Erlangen-Nürnberg (FAU), University Hospital Erlangen, Ulmenweg 18, 91054 Erlangen, Germany; 2 Institute of Medical Informatics, Biometry and Epidemiology, Friedrich-Alexander-Universität Erlangen-Nürnberg (FAU), Universitätsstraße 22, 91054 Erlangen, Germany; 3 Department of Urology and Pediatric Urology, Friedrich-Alexander-Universität Erlangen-Nürnberg (FAU), University Hospital Erlangen, Rathsberger Straße 57, 91054 Erlangen, Germany; 4 Department of Radiation Oncology, Friedrich-Alexander-Universität Erlangen-Nürnberg (FAU), University Hospital Erlangen, Universitätsstraße 27, 91054 Erlangen, Germany; 5 Department of Obstetrics and Gynaecology, Friedrich-Alexander-Universität Erlangen-Nürnberg (FAU), University Hospital Erlangen, Universitätsstraße 21/23, 91054 Erlangen, Germany; 6 Institute of Medical Physics, Friedrich-Alexander-Universität Erlangen-Nürnberg (FAU), Henkestraße 91, 91052 Erlangen, Germany

**Keywords:** Advanced cancer, Cancer cachexia, Nutrition, Physical exercise, Skeletal muscle mass, WB-EMS whole-body electromyostimulation

## Abstract

**Background:**

Physical exercise and nutritional treatment are promising measures to prevent muscle wasting that is frequently observed in advanced-stage cancer patients. However, conventional exercise is not always suitable for these patients due to physical weakness and therapeutic side effects. In this pilot study, we examined the effect of a combined approach of the novel training method whole-body electromyostimulation (WB-EMS) and individualized nutritional support on body composition with primary focus on skeletal muscle mass in advanced cancer patients under oncological treatment.

**Methods:**

In a non-randomized controlled trial design patients (56.5% male; 59.9 ± 12.7 years) with advanced solid tumors (UICC III/IV, *N* = 131) undergoing anti-cancer therapy were allocated to a usual care control group (*n* = 35) receiving individualized nutritional support or to an intervention group (*n* = 96) that additionally performed a supervised physical exercise program in form of 20 min WB-EMS sessions (bipolar, 85 Hz) 2×/week for 12 weeks. The primary outcome of skeletal muscle mass and secondary outcomes of body composition, body weight and hand grip strength were measured at baseline, in weeks 4, 8 and 12 by bioelectrical impedance analysis and hand dynamometer. Effects of WB-EMS were estimated by linear mixed models. Secondary outcomes of physical function, hematological and blood chemistry parameters, quality of life and fatigue were assessed at baseline and week 12. Changes were analyzed by t-tests, Wilcoxon signed-rank or Mann-Whitney-U-tests.

**Results:**

Twenty-four patients of the control and 58 of the WB-EMS group completed the 12-week trial. Patients of the WB-EMS group had a significantly higher skeletal muscle mass (0.53 kg [0.08, 0.98]; *p* = 0.022) and body weight (1.02 kg [0.05, 1.98]; *p* = 0.039) compared to controls at the end of intervention. WB-EMS also significantly improved physical function and performance status (*p* < 0.05). No significant differences of changes in quality of life, fatigue and blood parameters were detected between the study groups after 12 weeks.

**Conclusions:**

Supervised WB-EMS training is a safe strength training method and combined with nutritional support it shows promising effects against muscle wasting and on physical function in advanced-stage cancer patients undergoing treatment.

**Trial registration:**

ClinicalTrials.gov NCT02293239 (Date: November 18, 2014).

## Background

A high percentage of patients with solid cancer undergoing oncological treatment experiences weight loss in line with a progressive decline in skeletal muscle mass, a hallmark of cancer cachexia [1, 2]. Especially muscle loss is responsible for a poorer tolerance and greater complications during therapy resulting in higher progression and mortality rates of affected patients [3–7]. Systemic inflammatory activities increase catabolic processes and therapy-related gastrointestinal side effects, and a fatigue-induced low physical activity enhances the patients’ wasting, leading to an impaired quality of life [8–11]. The multi-factorial character of this syndrome challenges researchers to develop suitable and efficient counteracting measures and emphasizes the need for multimodal approaches [12]. The muscle building effects and the functional benefits of physical exercise are well known and even supported by cancer studies that show improvements in body composition, physical function and inflammation [13–16]. However, most studies included early-stage breast and prostate cancer patients, while trials with advanced cancer patients are scarce due to their decreased ability to perform conventional strength training [13, 17]. A time-saving and easy to-carry-out option may be provided by the novel training method of whole-body electromyostimulation (WB-EMS). Its application includes mild physical exercises and enables a simultaneous activation of almost all large muscles by electrodes integrated in a vest and belts. The efficacy of WB-EMS in increasing muscle strength and mass in athletes [18], was also demonstrated in elderly, sedentary persons and patients with chronic heart failure [19–24], but studies with cancer patients are still lacking. Regular exercise requires an adequate nutrient and energy supply to enable muscle growth and prevent muscle degradation. Due to the catabolic burden of cancer patients, dietary guidelines suggest a high daily protein intake of 1.0–2.0 g/kg body weight to cover individual protein requirements [25, 26]. However, a balanced protein turnover could only be demonstrated as a short-term effect of high amino acid intake, but results were unsatisfying in long-term studies, especially in advanced cancer [27, 28]. A better anabolic efficacy is suggested when resistance training is combined with protein supplementation [29], a finding that was also demonstrated in a cachectic tumor rat model [30]. Thus, a superior benefit of an approach combining physical exercise and nutrition in cancer-related muscle loss is obvious, but studies addressing this thesis are still missing.

The present pilot study reports on the effect of a combined intervention of individualized nutritional support and WB-EMS on skeletal muscle mass in advanced-stage cancer patients undergoing treatment. We hypothesized that this dual therapy has a stronger impact on stabilizing the skeletal muscle mass, our primary outcome, than nutrition alone. Effects on body weight and composition, physical function and performance, hematological parameters and blood chemistry, patient-reported quality of life and fatigue were also analyzed.

## Methods

### Patients

Patients (≥ 18 years) diagnosed with advanced solid tumors (UICC stage III and IV), an ongoing anti-cancer therapy and a Karnofsky performance index between 60 and 100 were considered as eligible for study inclusion after they declared their written informed consent to participate. The protocol of the study was approved by the Ethics Committee of the Friedrich-Alexander-Universität Erlangen-Nürnberg (FAU) (Registration No.155_13B) and retrospectively registered at clinicaltrials.gov (NCT02293239; November 13, 2014). During November 2013 and March 2016 cancer patients were recruited from different departments of the University Hospital Erlangen treating oncological patients. WB-EMS training, assessments and data collection were conducted in the Department of Medicine 1 – Gastroenterology, Pneumology and Endocrinology of the University Hospital Erlangen. Patients were excluded due to concomitant study inclusion in other nutritional or physical exercise trials, recently occurred acute cardio-vascular events, intake of anabolic drugs, pregnancy, epilepsy, severe neurological diseases, skin lesions within the area of electrodes, oncological surgery in the last 3 months, acute vein thrombosis, and the presence of cardiac pacemaker or conductive implants that would affect WB-EMS application.

### Study design

The pilot study was conducted as a two-armed prospective controlled clinical trial. After baseline assessment, patients were allocated to a physical exercise group performing a regular WB-EMS training (WB-EMS group). Patients who were interested in the study, but could not attain the WB-EMS training twice a week for 12 weeks due to a very long journey to the study center, were asked to join the control group. Thereby, special attention was on balanced patients’ characteristics. Both groups received nutritional support during the study. For each patient the duration of the study intervention was 12 weeks; in the WB-EMS group the outcome measure of body composition and physical function tests were conducted 1 week after the last training session to allow muscle regeneration. Due to the type of study intervention neither a blinding of the patients nor of the persons administering the intervention and assessing the outcome was done.

### Dietary support

In both groups nutritional intake was monitored by 24 h-dietary records (Freiburger Ernährungsprotokoll; Nutri-Science GmbH, Freiburg) assessed on 3 days in a row at study entry and within the last week of the study intervention. During the trial, patients documented their nutritional intake for 1 day per week. Computer-based analysis of mean caloric and nutrient intake was done by Prodi®6 expert (Nutri-Science GmbH, Freiburg). Based on these data individual dietary advices were given by a dietician at the beginning of the trial by face-to-face conversation. Then, nutritional intake of both study groups was controlled every fourth week in line with the other intermediate measures. Patients were advised to achieve a daily protein intake of > 1.0 g/kg following current dietary recommendations for patients with malignant disease undergoing anti-cancer treatment [25, 26]. Total energy intake was calculated according to the resting energy requirement (using Harris-Benedict equation) [31], physical activity level and nutritional status. A minimum energy intake of 25 kcal/kg/d was intended [25]. Patients showing renal failure were instructed not to exceed a daily protein intake of 1.0 g/kg in acute or 1.2 g/kg in chronic disease [32]. Nutritional intake for overweight persons (BMI ≥ 25 kg/m^2^) was adjusted to their normal weight calculated by the Broca Index (= height [cm] - 100) to prevent excessive calorie and protein consumption. In regard to usual care routine, patients whose food intake is reduced due to cancer cachexia, side effects of pharmacological treatment and/or gastrointestinal disorders were supported by supplemental nutrition in form of protein−/amino acid-rich oral supplements, or enteral or parenteral nutrition (Olimel 5.7% E, Baxter Germany, Munich). At baseline, the nutritional risk of the study patients was documented by nutritional risk screening 2002 (NRS 2002) classifying patients with a score of ≥3 to be at nutritional risk [33].

### Physical exercise program

The intervention group with physical exercise performed a WB-EMS training twice a week for a period of 12 weeks (a total number of 24 trainings). To allow muscle recovery a break of at least 2 days between each session was scheduled. Patients were adapted to WB-EMS by an initial training duration of 12 min/session that was progressively increased by 2 min per week up to the target time of 20 min/session. This was maintained until the last training session. WB-EMS training included light dynamic physical exercises according to a video tutorial and each patient was individually supervised by certified trainers. Seven different dynamic exercises - each repeated six times over a period of 1 min - were performed in a successive order (Table 1).

**Table 1:** Exercises performed during WB-EMS application

Exercises during WB-EMS application	
1. Squat combined with retroversion of extended arms	
2. Squat combined with butterfly-exercise of arms	
3. Lunge combined with anteversion of arms	
4. Lunge combined with retroversion of inflected arms	
5. Upright standing position combined with minimal forward bend and inner/outer arm rotation	
6. Squat combined with oblique forward bend	
7. Squat combined with horizontal abduction of arms	

Light dynamic exercises supported the muscle activation by WB-EMS

Abbreviations: *WB-EMS* Whole-body electromyostimulation

Selected exercises were light motions that support the muscle activation of upper limbs, lower limbs, gluteal and back muscles. The exercises are very easy to perform and suitable even for physically weakened persons. Exercises were only added to sustain the muscle activating effect of the WB-EMS and were adjusted to the patients’ ability in performing the motion sequence. The WB-EMS protocol was adapted to previous studies by Kemmler et al. [19, 20]. Electric muscle stimulation was applied by bipolar impulses at a frequency of 85 Hz and a pulse width of 350 μs inducing a 6 s muscle stimulation followed by a 4 s resting time. During the first WB-EMS application, the current intensity was set to generate a noticeable but comfortable muscle contraction; subsequently the current intensity was increased up to a threshold between being comfortable and inducing discomfort/pain. During WB-EMS patients wore a vest, a hip belt and upper-arm and -thigh cuffs with integrated electrodes to mediate the stimulation of muscles of the upper legs, the upper arms, the bottom, the abdomen, the chest, the upper and lower back and the large back muscle as described in detail by Kemmler et al. [21]. Equipment was used from Miha bodytec (Miha bodytec GmbH, Gersthofen).

### Study outcomes

The primary outcome was the change of skeletal muscle mass assessed by bioelectrical impedance analysis - BIA (seca mBCA 515; Seca GmbH & Co. KG, Hamburg). BIA was conducted at baseline and after 4, 8 and 12 weeks of study entry. Skeletal muscle mass calculated by BIA was shown to positively correlate with muscle mass determined by magnetic resonance imaging in healthy adults [34]. Secondary endpoints of body composition included body weight, fat mass percentage, ratio of extracellular fluid to intracellular fluid (hydration) and phase angle that were also assessed by BIA at the indicated time points.

Further secondary outcomes included physical function (hand grip strength, six-minute walking distance), performance status, quality of life and fatigue. During the visits at baseline and after 4, 8 and 12 weeks of study entry, isometric hand grip strength was measured by a hydraulic hand dynamometer (SH5001, SAEHAN Corporation, Masan, South Korea) in kg with a precision of 0.5 kg [35]. Patients were instructed to apply maximal power to the dynamometer with elbow flexed at 90°, with 3 repetitions for each hand. The maximum value of the 3 measurements was noted and for outcome calculations hand grip strength of the dominant hand was used. Functional capacity and endurance of the study patients were assessed by the six-minute walk test at baseline and week 12 [36]. Performance status was determined by the Karnofsky Index [37].

The psycho-social status of the patients was assessed by questionnaires at baseline and after 12 weeks. Thereby, the quality of life of the study participants was evaluated by the European Organisation for Research and Treatment of Cancer Quality of Life Questionnaire - C30 (EORTC QLQ-C30) [38]. For a precise assessment of fatigue the Functional Assessment of Chronic Illness Therapy - Fatigue Scale (13-item FACIT Fatigue Scale) was used [39].

The exercise adherence rate of the patients who finished the 12-week intervention period was calculated using the number of performed trainings in relation to the intended number of 24 WB-EMS trainings.

### Analysis of blood samples

Blood samples were collected at baseline and after 12 weeks of the study intervention either by puncture of the arm vein or corresponding blood values were extracted from the documented routine blood sampling undertaken during the oncological treatment. The analysis for inflammatory and nutritional blood markers (C-reactive protein, CRP, normal value < 5 mg/l; albumin, 35–55 g/l; total protein, 66–83 g/l), parameter of renal function (creatinine, 0.51–1.17 mg/dl) and hematological parameters (leucocytes, 4.4–11.3 × 10^3^/μl, thrombocytes 150–300 × 10^3^/μl, hematocrit 35–48%, hemoglobin, 11.5–18.0 g/dl, erythrocytes 4.1–6.0 × 10^6^/μl) was done by the diagnostic laboratories of the University Hospital Erlangen.

### Measurement of muscle damage and renal function parameters

In order to assess the degree of muscle damage, muscle enzymes including creatine kinase (CK; normal value: < 190 IU/l), myoglobin (Mb; < 70 μg/l), lactate dehydrogenase (LDH; < 250 U/l), AST (< 50 U/l), ALT (< 50 U/l), and indicators of renal functioning including potassium (3.7–5.5 mmol/l), sodium (146–157 mmol/l), inorganic phosphate (2.5–4.5 mmol/l), urea (17–43 mg/dl), uric acid (2.4–7.0 mg/dl), osmolality (280–300 mosmol/kg), creatinine (0.51–1.17 mg/dl) and glomerular filtration rate (GFR estimated by MDRD formula; > 60 ml/min) were measured at weeks 0, 2, 4, 8 and 12 in a subset of the WB-EMS group [40, 41]. To evaluate if blood parameters decline to baseline levels after 12 weeks of WB-EMS intervention, an additional blood analysis was done at week 13 conducted by the Central Laboratory of the University Hospital Erlangen.

### Statistical analysis

Data for baseline comparison of the two study groups are presented as total numbers and percentages or as mean ± standard deviation (SD). Blood parameters are presented as median and range (minimum to maximum). Based on the test for normal distribution (Shapiro-Wilk test) group differences of continuous variables were analyzed by independent samples t-test or Mann-Whitney-U-test. Categorical variables were analyzed by Pearson’s Chi-squared test.

With regard to the relatively high dropout rates or missed visits in our study resulting in missing data, we used linear mixed models (LMM) to estimate the effects of WB-EMS on body composition parameters and hand grip strength over time compared to the control group. LMM do not need the data of each visit from every individual and thus allow the inclusion of recruited patients even though a visit was missed during the study period of 12 weeks without the need of imputation [42, 43]. So no imputation for missing data was applied in this study. We fitted a LMM to each outcome using a random intercept for every individual. For each outcome the baseline value was included in the model as well as the variable time and a time-group interaction to estimate the intervention effect on the study outcome over time. The estimated effects are presented as mean difference between study groups and 95% confidence intervals and estimated marginal means with SEM. Secondary outcomes of quality of life, fatigue, physical function and performance as well as blood chemistry and hematology were analyzed for patients with assessments at baseline and after 12 weeks of intervention by paired samples t-test or the non-parametric Wilcoxon signed-rank test for within-group changes. Independent samples t-test or Mann-Whitney-U-test were used to compare differences in baseline to 12-week post-intervention changes between the study groups. Due to the exploratory character of this pilot study, no correction for multiple testing was applied.

To evaluate the effect of WB-EMS training on serum indicators of muscle damage and renal function during the study course, the Friedman test for non-parametric repeated measures comparisons followed by the Dunn’s multiple comparison post-hoc test was applied. Correlation analysis was carried out by the Spearman rank correlation.

Statistical analysis was conducted in R version 3.3.1 (R Foundation for Statistical Computing, Vienna, Austria), SPSS version 21 (IBM SPSS Statistics, Ehningen, Germany) and GraphPad Prism 6.07 (GraphPad Software Inc., La Jolla, CA, USA). Results were considered as statistically significant when *p* ≤ 0.05.

## Results

### Participants

**Fig. 1:**
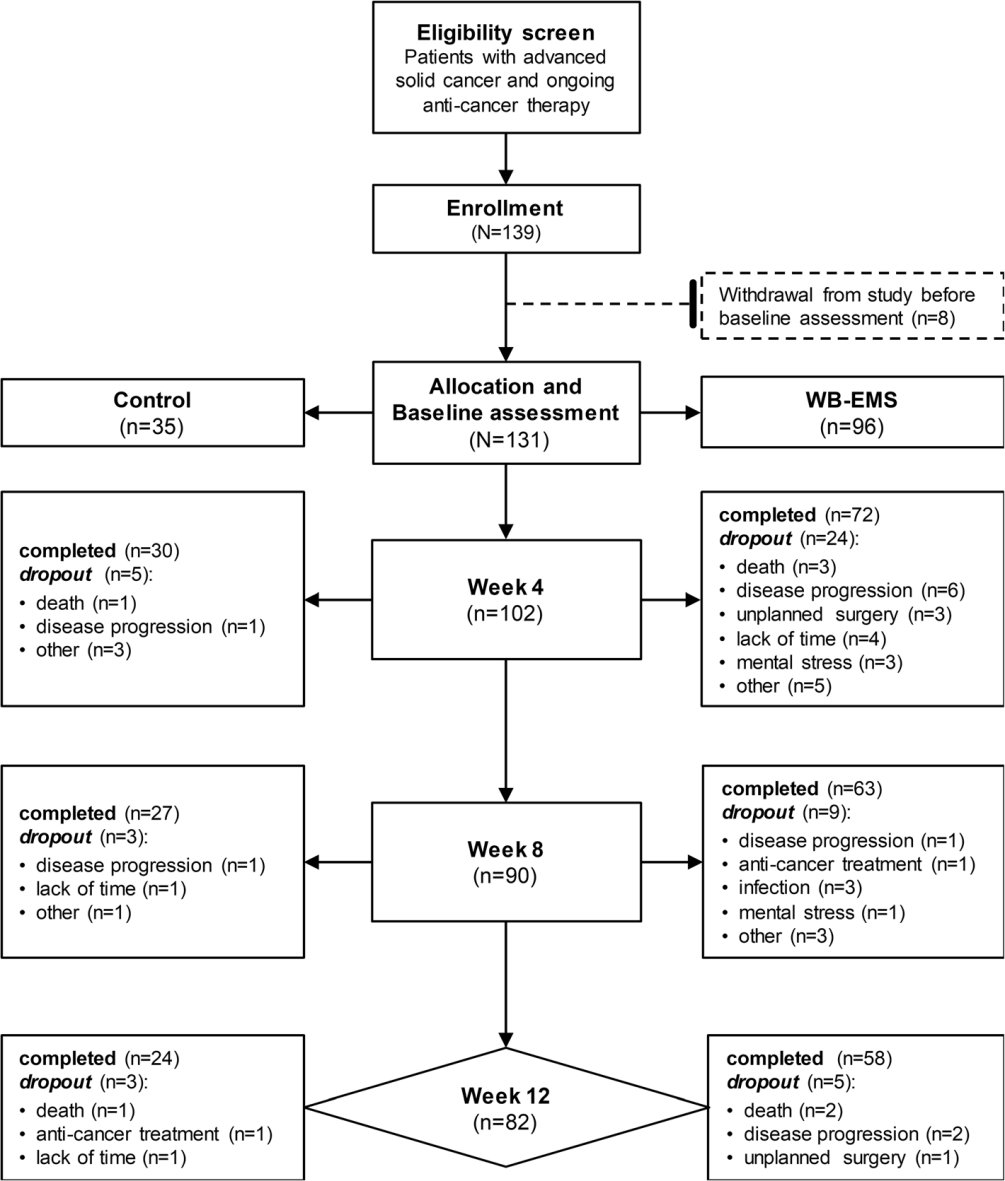
Patient flowchart. The flowchart shows the number of recruited and allocated patients, the number of patients who refused to participate before baseline assessment, the number of patients who dropped out during study course mentioning specific reasons and the number of patients that completed the whole intervention period of 12 weeks. Abbreviations: WB-EMS, whole-body electromyostimulation

A total of 139 patients were recruited after eligibility screening. Eight patients did not undergo baseline assessment due to fast and unexpected deterioration, death or disinterest. Thus, 131 participants were allocated to a control (*n* = 35) or WB-EMS group (*n* = 96) (Fig. 1). Patients’ characteristics are presented in Table 2.

**Table 2:** Demographic and disease characteristics of the included patients^b^

Parameter	WB-EMS	n	Control	n	*p*-value
Gender					0.270^c^
Male	57 (59.4%)		17 (48.6%)		
Female	39 (40.6%)		18 (51.4%)		
Age	60.3 ± 13.1	96	59.1 ± 11.6	35	0.541^d^
Functional status					
Performance status [Karnofsky index]	75.7 ± 10.1	96	76.6 ± 10.0	35	0.517^d^
Hand grip strength [kg]	36.5 ± 12.0	88	38.8 ± 15.0	33	0.616^d^
Six-minute walking distance [m]	505.7 ± 118.0	78	483.1 ± 126.8	31	0.378^e^
Nutritional status		96		35	
Body weight [kg]	74.4 ± 14.9		74.9 ± 16.9		0.773^d^
BMI [kg/m^2^]	24.9 ± 3.8		25.5 ± 5.2		0.707^d^
Fat mass [%]	29.6 ± 7.5		29.9 ± 8.6		0.833^e^
Fat free mass index [kg/m^2^]	17.5 ± 2.6		17.4 ± 3.2		0.864^e^
Phase angle [°]	4.5 ± 0.8		4.5 ± 0.7		0.898^e^
Hydration [%]	86.4 ± 11.5		86.3 ± 9.2		0.970^d^
Skeletal muscle mass [kg]	23.5 ± 6.0		23.6 ± 7.0		0.950^d^
Nutritional risk screening					0.210^c^
< 3	35 (36.5%)		17 (48.6%)		
≥ 3	61 (63.5%)		18 (51.4%)		
Nutritional therapy					0.539^c^
Only dietary counseling	65 (67.7%)		21 (60.0%)		
Oral supplementation	25 (26.0%)		11 (31.4%)		
Feeding tube	2 (2.1%)		0 (0.0%)		
Parenteral	4 (4.2%)		3 (8.6%)		
Mean daily nutrient/caloric intake during participation [per kg body weight]		89		29	
Carbohydrates [g]	3.4 ± 1.2		3.4 ± 1.5		0.567^d^
Fat [g]	1.2 ± 0.5		1.3 ± 0.6		0.841^d^
Protein [g]	1.3 ± 0.6		1.3 ± 0.5		0.604^d^
Calories [kcal]	30.9 ± 9.3		32.1 ± 13.1		0.988^d^
Disease characteristics					
Cancer sites					
Head and Neck	4 (4.2%)		0 (0.0%)		
Esophagus	1 (1.0%)		2 (5.7%)		
Stomach	9 (9.4%)		1 (2.9%)		
Colon	13 (13.5%)		8 (22.9%)		
Rectum	6 (6.3%)		5 (14.3%)		
Pancreas	5 (5.2%)		7 (20.0%)		
Liver	3 (3.1%)		1 (2.9%)		
Bile duct	2 (2.1%)		1 (2.9%)		
Lung	15 (15.6%)		3 (8.6%)		
Prostate	11 (11.5%)		0 (0.0%)		
Kidney	7 (7.3%)		0 (0.0%)		
Breast	12 (12.5%)		4 (11.4%)		
Ovary	3 (3.1%)		1 (2.9%)		
Others	5 (5.2%)		2 (5.7%)		
UICC stage					0.165^c^
III	28 (29.2%)		6 (17.1%)		
IV	68 (70.8%)		29 (82.9%)		
Ongoing anti-cancer therapy					0.082^c^
Chemotherapy	46 (47.9%)		21 (60.0%)		
Radiotherapy	7 (7.3%)		0 (0.0%)		
Chemoradiation	3 (3.1%)		0 (0.0%)		
Targeted therapy	14 (14.6%)		1 (2.9%)		
Hormonal therapy	6 (6.3%)		1 (2.9%)		
Chemotherapy + Targeted therapy	11 (11.5%)		10 (28.6%)		
Chemotherapy + Hormonal therapy	4 (4.2%)		1 (2.9%)		
Radiotherapy + Hormonal therapy	3 (3.1%)		0 (0.0%)		
Others	2 (2.1%)		1 (2.9%)		
Previous cancer-related surgery	68 (70.8%)		20 (57.1%)		0.140^c^
Number of current medications	3.3 ± 2.7	96	3.8 ± 2.6	35	0.328^d^
Comorbidities^a^					
Cardiovascular disease	30 (31.3%)		7 (20.0%)		0.206^c^
Thyroid disease	27 (28.1%)		10 (28.6%)		0.960^c^
Pulmonary disease	20 (20.8%)		11 (31.4%)		0.230^c^
Diabetes mellitus	12 (12.5%)		4 (11.4%)		0.838^c^
Renal failure	5 (5.2%)		2 (5.7%)		0.919^c^
Liver disease	3 (3.1%)		3 (8.8%)		0.187^c^
Pancreatitis	2 (2.1%)		1 (2.9%)		0.800^c^
Laboratory values					
Erythrocytes [×10^6^/μl]	4.18 (2.62–5.30)	85	4.18 (3.48–5.10)	32	0.176^e^
Hemoglobin [g/dl]	12.7 (7.7–15.0)	85	12.3 (8.8–15.4)	32	0.588^d^
Hematocrit [%]	37.1 (22.8–45.7)	85	36.5 (28.2–44.8)	32	0.739^e^
Leucocytes [× 10^3^/μl]	5.1 (1.2–18.9)	85	5.8 (3.1–8.8)	32	0.349^d^
Thrombocytes [× 10^3^/μl]	209.0 (84.0–1014.0)	85	198.0 (74.0–444.1)	32	0.342^d^
Creatinine [mg/dl]	0.85 (0.47–1.41)	81	0.82 (0.51–1.01)	30	0.120^e^
Total protein [g/l]	66.3 (49.9–80.5)	65	68.5 (39.7–82.6)	22	0.232^d^
Albumin [g/l]	40.7 (26.7–48.1)	66	41.7 (28.4–46.7)	22	0.528^d^
CRP [mg/l]	5.2 (0.2–155.2)	78	5.2 (1.0–48.9)	30	0.880^d^

Abbreviations: *CRP* C-reactive protein, *CUP* Cancer of unknown primary, *WB-EMS* Whole-body electromyostimulation

^a^Note: Number of patients for comorbidities includes also patients with several comorbidities

^b^Data are presented in numbers and proportions (%) and as mean ± standard deviation. Laboratory values are expressed as median and range (minimum to maximum value). Statistically significant differences are indicated by *p* ≤ 0.05

^c^Pearson’s Chi-squared test

^d^Mann-Whitney-U-test

^e^Independent samples t-test

Baseline assessment showed no significant group differences in cancer stage and treatment regimens, number of medications or comorbidities. Patients of different cancer sites were recruited with highest proportions of gastrointestinal, gynecological and lung cancer in both study groups. Due to the large variety of different cancer sites, no *p*-value is presented here. Patients of both study groups were not significantly different in the functional status at baseline indicated by a comparable Karnofsky performance status and physical function tests (six-minute walk test, hand grip strength). Participants of control and WB-EMS group showed normal body mass indices at baseline. However, body composition was characterized by a high fat mass percentage and low fat free mass index [44]. Initial values of skeletal muscle mass were not different between control and WB-EMS group. Low phase angle values in the WB-EMS and the control group indicated for impaired nutritional and functional status of cancer patients [45]. Approximately 60% of the participants in the WB-EMS group and 50% of the patients in the control group were at nutritional risk at study entry as indicated by a score ≥ 3 points assessed by the nutritional risk screening (NRS) [45].

During the study period the mean daily protein intake was > 1.0 g/kg and the mean caloric intake > 30.0 kcal/kg for all included patients with available nutritional data in both study groups without significant differences (Table 2). A more detailed evaluation of the nutrient intake of each group revealed that 32.6% and 31.0% of the participants in the WB-EMS and the control group, respectively, showed a protein intake of ≤1.0 g/kg/d. A high daily protein intake of > 1 g/kg to 1.5 g/kg was achieved by 44.9% of the patients in both study groups. Very high protein intake of > 1.5 g/kg was achieved by 22.5% of the patients in the training group and by 24.1% of the controls. No significant differences between the two study groups were observed regarding the different level of protein consumption (*p* = 0.979). A daily caloric intake of < 25 kcal/kg was seen in 25.8% and 24.1% of the WB-EMS and the control participants, respectively. Caloric intake of 25–30 kcal/kg/d was achieved by 27.0% of the WB-EMS patients and by 31.0% of the controls. Higher daily caloric intake of > 30 kcal/kg was observed for 47.2% and 44.8% of the participants in the training and the control group, respectively. No significant group differences for caloric intake level were detected (*p* = 0.914). The percentage of patients with inadequate nutrient intake who had to be supported by enteral or parenteral nutrition in regard to usual care guidelines was balanced between the study groups.

At baseline, no significant differences in blood parameters were observed (Table 2).

During the trial, 29, 12 and 8 participants withdrew from the study at weeks 4, 8 and 12, respectively (Fig. 1). Premature dropout was due to disease progression (*n* = 11), death (*n* = 7), lack of time (*n* = 6), unplanned surgery (*n* = 4), mental stress (n = 4), infection (*n* = 3), strong side effects of anti-cancer treatment (*n* = 2) and other reasons (*n* = 12) (Fig. 1). Dropout reasons were not different between the study groups (*p* = 0.594). The dropout rate of the WB-EMS group was not significantly different from controls (39.6% and 31.4%, *p* = 0.393). No patient withdrew from the study due to discomfort or adverse events related to the WB-EMS training.

Patients from both groups who prematurely terminated the study (*n* = 49), had significantly higher serum CRP concentrations and leucocyte count, lower albumin, total protein and lower hemoglobin concentrations at baseline compared to patients who completed the study (CRP, 22.1 ± 31.0 mg/l vs. 8.4 ± 15.3 mg/l; *p* < 0.001; leucocyte count, 6.6 ± 3.4 × 10^3^/μl vs. 5.2 ± 1.7 × 10^3^/μl; *p* = 0.044; albumin, 38.4 ± 5.0 g/l vs. 40.7 ± 4.0 g/l; *p* = 0.025; total protein, 63.5 ± 8.3 g/l vs. 68.7 ± 5.5 g/l; *p* = 0.004; hemoglobin, 11.9 ± 1.6 g/dl vs. 12.5 ± 1.6 g/dl; *p* = 0.034). Compared to participants who finished the intervention period, dropped out patients by trend showed a more limited role (50.5 ± 32.3 vs. 59.8 ± 29.3; *p* = 0.137), cognitive (70.4 ± 27.1 vs. 78.9 ± 21.4; *p* = 0.102) and social functioning (48.4 ± 33.5 vs. 57.0 ± 29.2; *p* = 0.108). Further, they were characterized by a tendentially higher symptom level of dyspnoea (38.4 ± 39.0 vs. 24.1 ± 28.1; *p* = 0.081). The daily medication intake was also significantly higher in the dropped-out patients (4.2 ± 2.6) than in the patients who completed the trial (3.3 ± 2.6; *p* = 0.030).

At baseline, patients of the WB-EMS group who did not complete the final assessment showed significantly lower scores in cognitive functioning (66.6 ± 26.7 vs. 85.4 ± 24.3; *p* = 0.036) and by trend worse global health (49.5 ± 22.8 vs. 61.4 ± 16.7; *p* = 0.177) compared to the dropout patients of the control group. Parameters of performance status and body composition of the dropped-out patients of both groups did not differ at study entry.

During the trial, 7 participants of the WB-EMS and 6 participants of the control group missed the follow-up visit at week 4. Thus, 65 WB-EMS and 24 control patients were measured by BIA at this time point. At week 8, two patients of the WB-EMS and 6 patients of the control group missed the body composition assessment. Thus, 61 WB-EMS and 21 control patients were assessed by BIA at the third visit. A total of 82 patients completed the study period of 12 weeks, whereby 58 participants of the training group and 24 of the control group had the final outcome assessment. For one WB-EMS participant, the body composition parameters could not be determined after 12 weeks of intervention due to technical problems with the BIA measurement.

Participants of the WB-EMS group who completed the full 12-week intervention period underwent 20.8 ± 2.6 (range from 13 to 24 trainings) of the scheduled 24 training sessions leading to an adherence rate of 86.6 ± 10.9% (range from 54.2 to 100%).

Besides weak symptoms of muscle soreness no side effects of the WB-EMS training were observed.

### Body composition

**Fig. 2:**
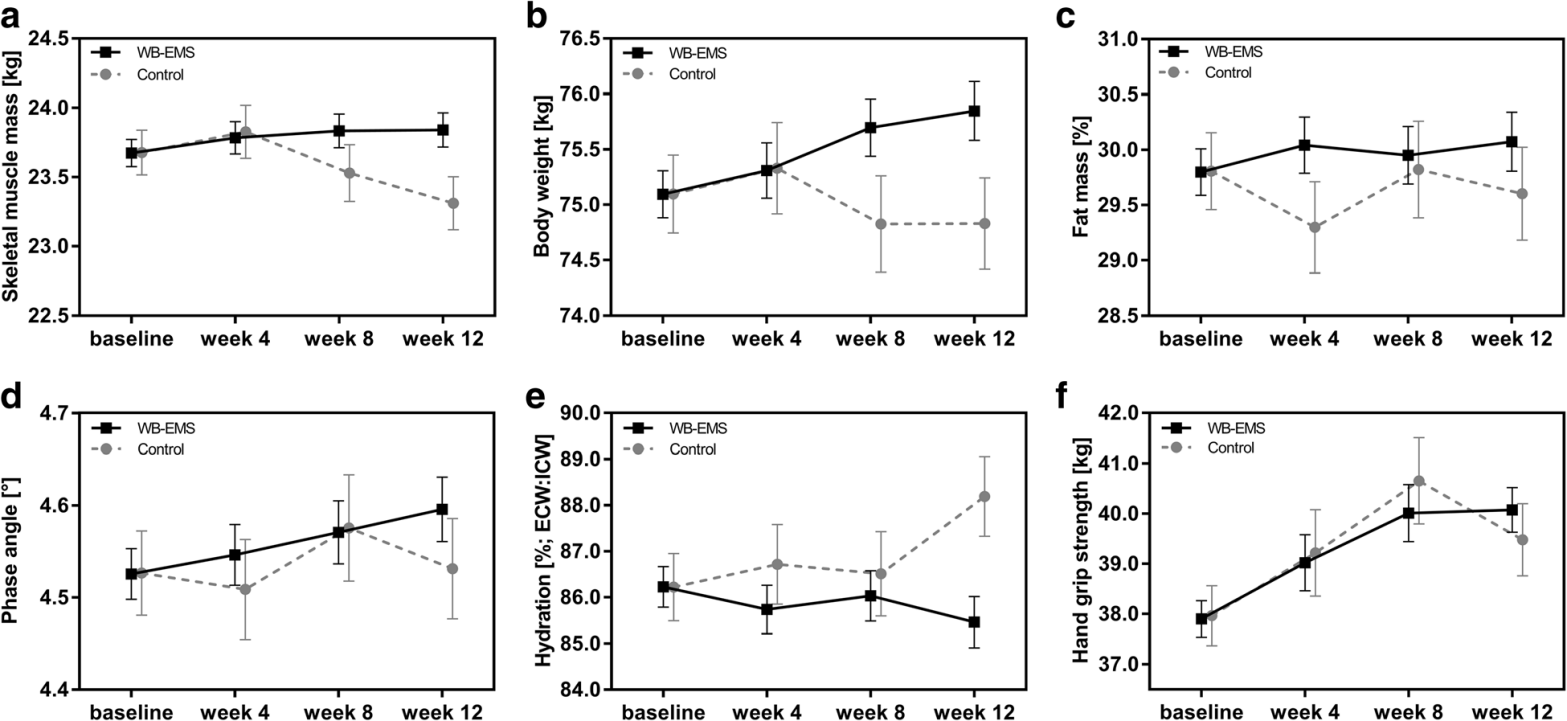
Estimated marginal means from LMM for body composition parameters and hand grip strength. **a**-**f** Estimated marginal means from linear mixed models (error bars show SEM) for body composition and hand grip strength are illustrated of the control group and the WB-EMS group at the different measuring times during the 12-week intervention period. Abbreviations: ECW, extracellular water; ICW, intracellular water; LMM, linear mixed models; WB-EMS, whole-body electromyostimulation

Figure 2 demonstrates the estimated marginal means calculated by the linear mixed models. Table 3 presents the estimated effects of the WB-EMS intervention compared to the control as mean difference between study groups with 95% confidence intervals for each measurement time.

**Table 3:** Estimated effect of WB-EMS on body composition and hand grip strength calculated by LMM

	Estimated effect of WB-EMS intervention compared to controls^a^					
	Week 4	*p*	Week 8	*p*	Week 12	*p*
	Estimate [95% CI]		Estimate [95% CI]		Estimate [95% CI]	
Body composition						
SMM [kg]	−0.04 [− 0.49, 0.40]	0.848	0.31 [− 0.16, 0.78]	0.201	0.53 [0.08, 0.98]	**0.022**
Bodyweight [kg]	−0.02 [− 0.97, 0.93]	0.966	0.87 [− 0.13, 1.87]	0.077	1.02 [0.05, 1.98]	**0.039**
FM [%]	0.75 [−0.20, 1.70]	0.121	0.14 [−0.87, 1.14]	0.789	0.51 [−0.46, 1.47]	0.302
PhA [°]	0.04 [−0.09, 0.016]	0.557	−0.01 [− 0.14, 0.13]	0.946	0.07 [− 0.06, 0.19]	0.320
Hydration [%]^b^	− 0.98 [− 2.97, 1.01]	0.334	− 0.48 [− 2.58, 1.62]	0.655	− 2.73 [− 4.76, − 0.71]	**0.008**
Functional status						
Hand grip strength [kg]	−0.20 [− 2.24, 1.84]	0.847	− 0.64 [− 2.68, 1.39]	0.535	0.60 [− 1.08, 2.27]	0.484

Statistically significant effects are marked in bold type and indicated by *p* < 0.05

Abbreviations: *WB-EMS* Whole-body electromyostimulation, *SMM* Skeletal muscle mass, *FM* Fat mass, *PhA* Phase angle, *ECW* Extracellular water, *ICW* Intracellular water

^a^Linear mixed model analysis estimating the effect (group x time) of the combined WB EMS and nutrition intervention on the primary outcome of skeletal muscle mass and secondary outcomes of body composition and hand grip strength over the 12-week study course compared to the usual care control group. Data are presented as estimated mean difference between study groups and 95% confidence intervals [95% CI]

^b^Hydration represents ECW:ICW in %

During the trial, skeletal muscle mass increased in the WB-EMS group (estimated marginal means ± SEM; Fig. 2a). In the control, the patients skeletal muscle mass tended to increase at week 4, but dropped below baseline values at week 8 and 12 (Fig. 2a). After 12 weeks a significantly higher skeletal muscle mass of 0.53 kg was estimated via LMM for the WB-EMS group compared to controls (Table 3). The changes of skeletal muscle mass were reflected by alterations in body weight (Fig. 2b). Comparing the changes in body weight between the groups a significant estimated effect of 1.02 kg was observed favoring the WB-EMS group. Fat mass percentage did not show a distinct change in the control group, while in the WB-EMS group a moderate increase during the intervention period was detected (Fig. 2c). To assess health and nutrition status the phase angle was measured by BIA. The WB-EMS group showed a steady increase in phase angle, while it remained unchanged in the control group after 12 weeks (Fig. 2d). However, the estimated effect of WB-EMS on the phase angle was not statistically significant compared to controls (Table 3). In line with the phase angle analysis, changes in the hydration status assessed by BIA were observed. Hydration reflects the distribution between extra- and intracellular water and thus, significantly negatively correlated with phase angle (Spearman rank correlation, *r*_*s*_ = 0.901; *p* < 0.0001). The hydration status (increase of extracellular fluid compared to intracellular fluid) tended to increase in controls and decreased in the WB-EMS group (Fig. 2e). After 12 weeks of intervention WB-EMS patients showed a significantly lower hydration status in contrast to controls (Table 3).

### Isometric hand grip strength, physical function and performance status

Within the study period of 12 weeks, participants of the control and the WB-EMS group increased in hand grip strength (Fig. 2f). Although WB-EMS training seems to have a more pronounced effect on hand grip strength compared to the untrained controls, the model did not reveal a statistically significant effect (Table 3). However, physical function evaluated by walking distance (six-minute walk test) was significantly improved after 12 weeks in the WB-EMS group compared to the control group (Table 4) even though both study groups showed significant increases in the walking distance. Furthermore, performance status assessed by the Karnofsky Index showed a significant increase within the WB-EMS group after 12 weeks. This change was significantly higher compared to the control group (Table 4).

**Table 4:** Physical function and performance status of the study groups at baseline and week 12^a^

Outcome Measure	Study group	n	Baseline	Week 12	Difference week 12	*p* ^b^	*p* ^c^
Physical function							
SMWT distance [m]	Control	21	484.30 ± 135.0	504.6 ± 116.8	20.3 ± 48.2	**0.024**	**0.036**
	WB-EMS	51	521.6 ± 104.5	577.1 ± 95.4	55.4 ± 75.2	**< 0.001**	
Performance status							
Karnofsky Index	Control	24	75.4 ± 10.6	73.8 ± 12.1	−1.7 ± 10.5	0.412	**0.025**
	WB-EMS	57	76.4 ± 10.9	81.6 ± 12.4	5.2 ± 11.7	**0.002**	

Statistically significant differences are marked in bold type and indicated by *p* < 0.05

Abbreviations: *SMWT* Six-minute walk test, *WB-EMS* Whole-body electromyostimulation

^a^Includes patients with both baseline and post-intervention data. Data are presented as mean ± SD

^b^Comparison of intragroup differences in baseline and post-intervention data (Wilcoxon signed-rank test)

^c^Comparison of differences from baseline to 12-week post-intervention changes between the groups (Mann-Whitney-U-test)

### Hematological and blood chemistry parameters

Blood parameters of inflammation represented by albumin and CRP serum concentration were not statistically significantly affected by WB-EMS training after 12 weeks (paired samples t-test, albumin change 0.1 ± 3.1 g/l, *n* = 36; *p* = 0.904; Wilcoxon signed-rank test, CRP change − 2.5 ± 17.9 mg/l; *p* = 0.291; *n* = 45) and also did not significantly change in the control group (Wilcoxon signed-rank test, albumin change 0.8 ± 4.2 g/l, *p* = 0.460, *n* = 15; paired samples t-test, CRP change − 1.9 ± 7.6 mg/l; *p* = 0.122, *n* = 18). No significant differences between both groups were seen after 12 weeks (Mann-Whitney-U-test; albumin, *p* = 0.476; CRP, *p* = 0.411). Besides a significant reduction of erythrocyte count in the control group (paired samples t-test; erythrocyte count change − 0.2 ± 0.4 × 10^6^/μl; *p* = 0.045; *n* = 19), significant changes neither within nor between the study groups were detected in blood count and serum biochemistry parameters.

### Quality of life and fatigue

According to EORTC QLQ – C30 questionnaire participants of control group reported significant reduction in pain and fatigue and improved in global health (Table 5). Within the WB-EMS groups emotional functioning of participants was significantly ameliorated and social functioning was improved by trend. Symptoms of nausea and vomiting were significantly diminished. The more precise determination of fatigue by the FACIT-Fatigue Scale revealed a significant improvement of fatigue in both study groups, but statistical significance was only reached for the control group. No significant differences in changes of quality of life parameters and fatigue were observed between the two study groups after 12 weeks (Table 5).

**Table 5:** Fatigue and quality of life of the study groups at baseline and week 12^a^

Outcome Measure	Study group	n	Baseline	Week 12	Difference week 12	*p* ^b/c^	*p* ^d^
FACIT-Fatigue Scale	Control	18	33.11 ± 11.49	39.61 ± 7.69	6.50 ± 10.01	**0.014** ^c^	0.077
	WB-EMS	52	36.81 ± 10.76	38.58 ± 9.74	1.77 ± 8.62	0.058^b^	
EORTC QLQ-C30 Functional Scales							
Physical functioning	Control	19	74.37 ± 20.81	77.32 ± 15.51	2.95 ± 16.54	0.623^b^	0.542
	WB-EMS	55	78.55 ± 20.12	80.47 ± 20.90	1.93 ± 17.11	0.276^b^	
Role functioning	Control	17	54.94 ± 27.56	66.65 ± 22.86	11.71 ± 31.79	0.149^c^	0.242
	WB-EMS	55	63.62 ± 28.89	62.18 ± 30.15	− 1.44 ± 24.29	0.863^b^	
Emotional functioning	Control	19	63.53 ± 25.57	63.53 ± 25.86	0.00 ± 23.69	0.850^b^	0.096
	WB-EMS	53	66.62 ± 21.26	73.06 ± 21.41	6.43 ± 16.82	**0.007** ^**c**^	
Cognitive functioning	Control	19	80.63 ± 21.71	85.05 ± 19.95	4.42 ± 20.69	0.265^b^	0.791
	WB-EMS	53	78.00 ± 22.27	79.19 ± 24.01	1.19 ± 18.46	0.739^b^	
Social functioning	Control	19	50.89 ± 30.24	61.37 ± 28.88	10.47 ± 27.96	0.096^b^	0.501
	WB-EMS	53	58.89 ± 28.80	65.42 ± 28.42	6.53 ± 22.88	0.054^b^	
EORTC QLQ-C30 Symptom Scales							
Pain	Control	19	37.68 ± 30.81	16.58 ± 25.39	−21.11 ± 34.48	**0.016** ^**c**^	0.072
	WB-EMS	54	25.24 ± 26.41	21.89 ± 28.87	− 3.35 ± 26.34	0.254^b^	
Dyspnea	Control	18	27.78 ± 32.92	22.11 ± 27.98	−5.67 ± 26.20	0.196^b^	0.166
	WB-EMS	55	22.95 ± 27.13	27.82 ± 31.31	4.87 ± 25.98	0.156^b^	
Insomnia	Control	18	36.89 ± 30.07	29.61 ± 32.19	−7.28 ± 37.21	0.418^c^	0.606
	WB-EMS	55	28.45 ± 32.40	25.38 ± 29.40	−3.07 ± 35.39	0.379^b^	
Appetite loss	Control	18	40.72 ± 37.22	24.06 ± 32.00	− 16.67 ± 46.45	0.193^b^	0.051
	WB-EMS	55	21.20 ± 31.06	15.72 ± 28.59	−5.47 ± 27.81	0.119^b^	
Constipation	Control	19	22.84 ± 33.52	19.26 ± 30.08	−3.58 ± 35.12	0.619^b^	0.522
	WB-EMS	53	16.32 ± 28.97	14.40 ± 24.87	−1.92 ± 30.94	0.617^b^	
Diarrhea	Control	18	25.94 ± 29.39	25.94 ± 33.53	0.00 ± 25.70	1.000^b^	0.563
	WB-EMS	53	21.32 ± 27.87	17.58 ± 25.03	−3.74 ± 28.25	0.591^b^	
Financial difficulties	Control	19	22.79 ± 31.58	33.32 ± 38.54	10.53 ± 29.42	0.107^b^	0.206
	WB-EMS	52	21.79 ± 27.21	21.12 ± 28.83	− 0.67 ± 29.87	0.711^b^	
Nausea/Vomiting	Control	18	21.39 ± 24.06	15.72 ± 25.17	− 5.67 ± 37.00	0.524^c^	0.752
	WB-EMS	55	12.72 ± 17.17	6.69 ± 13.06	−6.04 ± 18.19	**0.032** ^**b**^	
Fatigue	Control	18	51.17 ± 22.49	33.89 ± 22.87	−17.28 ± 28.58	**0.020** ^**c**^	0.135
	WB-EMS	55	43.05 ± 28.23	39.96 ± 27.89	−3.09 ± 25.01	0.185^b^	
Global Health	Control	18	54.17 ± 19.52	66.67 ± 20.20	12.50 ± 24.77	**0.047** ^**c**^	0.157
	WB-EMS	53	59.40 ± 18.19	61.49 ± 20.78	2.09 ± 19.63	0.247^b^	

Statistically significant differences are marked in bold type and indicated by ^p^ < 0.05

Abbreviations: *WB-EMS* Whole-body electromyostimulation

^a^Includes patients with both baseline and post-test data. Data are presented as mean ± SD

^b^Comparison of intragroup differences in baseline and post-intervention data (Wilcoxon signed-rank test)

^c^Comparison of intragroup differences in baseline to 12-week post-intervention changes (paired samples t-test)

^d^Comparison of differences in baseline to 12-week post-intervention changes between the groups (Mann-Whitney-U-test)

### Muscle damage and renal function

To gather the effect of WB-EMS on serum indicators of muscle damage and their impact on renal functioning, a serial measurement of blood parameters was done in a subset of patients. Fifteen participants completed all measurements at the 6 scheduled time points and were therefore evaluated for repeated measures comparison; one patient was excluded from analysis due to severe chronically elevated myoglobin concentrations. Thus, 14 participants were analyzed, including 11 men and 3 women (61.1 ± 9.5 years) with different cancer sites (urological *n* = 7, gastrointestinal *n* = 6, lung *n* = 1) and advanced disease stages (UICC III/IV, *n* = 1/13). One patient had no first measurement of the Mb concentration, so data of Mb represent the values of 13 patients. As presented in Table 5, the range of CK value elevation in response to WB-EMS training showed strong individual differences. Over the study course WB-EMS intervention led to an elevation in CK values (Table 6). Multiple comparisons test between every time point revealed a significant mean 2.9-fold increase in serum concentrations of CK from baseline to 8 weeks of WB-EMS (*p* = 0.002). This 2.9-fold increase from baseline remained until week 12 (*p* = 0.043). One week after the last WB-EMS session, CK values were significantly decreased compared to the values of week 8 (*p* = 0.043) and only a mean non-significant 1.4-fold increase from baseline was observed. Serum concentrations of Mb were described to be elevated in response to resistance training and WB-EMS [40, 41]. Although the Mb serum levels did not differ significantly over the 12 weeks of our exercise intervention, the Mb concentration correlated positively with the CK elevation during the study course in a significant manner (*r*_*s*_ = 0.943, *p* = 0.017). Mb levels were also highest in week 8 and 12 (1.3-fold increase compared to baseline; Table 5). The concentration of other serum indicators of muscle damage including LDH, AST and ALT did not significantly change over the study course (data not shown).

**Table 6:** Serum indicators of muscle damage during the WB-EMS intervention period

Parameter	Study week						p-value^b^
	Baseline	week 2	week 4	week 8	week 12	week 13	
CK [U/l]^a^							
mean ± SD	104.2 ± 50.29	195.1 ± 204.4	209.9 ± 272.0	272.1 ± 211.4**	234.5 ± 181.5*	128.1 ± 66.5^†^	**0.002**
median	103.0	122.0	141.5	178.0	195.5	135.5	
range	33–192	34–817	39–1130	56–626	55–713	43–233	
Mb [μg/l]^a^							
mean ± SD	76.5 ± 15.8	92.5 ± 33.3	90.5 ± 30.2	100.4 ± 32.7	93.5 ± 34.0	81.4 ± 24.4	0.080
median	78.0	86.0	80.0	90.0	91.0	75.0	
range	42–102	52–182	42–166	73–190	62–187	48–136	

Statistically significant differences are marked in bold type and indicated by *p* < 0.05

Abbreviations: *CK* Creatine kinase, *Mb* Myoglobin, *WB-EMS* Whole-body electromyostimulation

^a^Normal values: CK < 190 U/l, Mb < 70 μg/l.

^b^Nonparametric repeated measures comparison over time (Friedman test) in the WB-EMS group (*n* = 14). CK values changed statistically significantly over time (*p* < 0.05) with significant differences of CK values at week 8 and 12 compared to baseline (Dunn’s multiple comparisons test; * *p* ≤ 0.05, ** *p* ≤ 0.01). One week after last WB-EMS session (week 13) CK values were significantly decreased compared to week 8 (^†^*p* ≤ 0.05). Data are presented as mean ± SD, median and range (minimum to maximum value)

Furthermore, repeated measures analysis of blood parameters specifying renal function and electrolytes did not show significant differences over the trial period (data not shown). No significant correlation between creatinine and elevated CK concentrations was observed (*r*_*s*_ = 0.314; *p* = 0.564). In addition, creatinine concentrations from a total of 48 patients with assessment at baseline and week 13 were unaffected by the WB-EMS intervention as analyzed by the paired sample t-test (mean ± SD; baseline 0.88 ± 0.19 mg/dl, after 13 weeks 0.88 ± 0.22 mg/dl; *p* = 0.800).

## Discussion

We here present the first study, to our knowledge, investigating the effect of strength training in form of WB-EMS combined with individualized nutritional support on advanced-stage cancer patients undergoing oncological treatment. The results of this pilot trial suggest WB-EMS combined with a protein-rich nutrition as safe and more effective in sustaining skeletal muscle mass than an exclusive dietary therapy that also potentially improves physical function and performance status in cancer patients.

Until now, only a few exercise studies analyzed skeletal muscle mass as a primary outcome in cancer patients and hardly any data are available for patients with advanced disease [46], who often show severe muscle wasting and malnutrition [4, 47], and thus physical weakness as a result of multiple catabolic processes and oncological therapy [3, 4, 48, 49]. In a systemic review by Stene et al., summarizing the effects of physical exercise on muscle status in cancer patients under treatment, 6 trials reported changes in muscle mass, but included only early-stage breast, prostate or hematological cancer [13, 50–54]. Two trials demonstrated an increase in the lean body mass in patients performing a combined aerobic/resistance training, and a decline in the usual care groups [52, 53]. Another trial reported a superior role of resistance training on lean body mass compared to aerobic exercise after a median of 17 weeks [54], while 3 trials did not reveal a significant effect [50]. These results suggest rather a maintaining than an increasing effect of exercise on muscle mass, at least in early-stage cancer patients. However, the studies did not address nutritional aspects, especially protein intake, as we did. In reference to those data, we were able to show that it is possible to induce a significantly higher muscle mass by combining the novel WB-EMS training technique with adapted nutrition, even in advanced cancer patients. Local EMS has already been tested for chronic benign diseases and is rated as safe and beneficial in improving muscle mass [55]. However, patients with advanced lung cancer did not benefit from this local, passive application form of EMS in a home-based setting [56]. We speculate that the systemic muscle breakdown in cancer cannot be counteracted by stimulating single muscles and that in contrast a supervised whole-body application seems to be more potent in increasing muscle mass and has a good exercise adherence rate (86.6%) in patients who were able to complete the trial. Remarkably, this adherence was higher than in another exercise study including patients with different advanced-stage cancers (69%) who had pre- and post-tests [57] and may be linked to the effortless and time-saving features of WB-EMS. Future studies should investigate if WB-EMS training may be more effective and suitable for advanced cancer patients than conventional strength techniques.

Physical weakness, fatigue, depression, therapeutic side effects and symptoms of disease progression are the main reasons for a premature withdrawal of cancer patients from exercise programs [11]. In our study, disease progression linked with a fast physical deterioration was the main cause of dropout. However, the termination rates of 39.6% in the exercise and 31.4% in the control group were comparable to other exercise trials reporting rates of 35% in the intervention and 22% in the usual care group for similar reasons [57]. As an explanation for higher dropout rates in the exercise group it may have been less burdensome for control patients to attend monthly intermediate measures than to regularly participate in an exercise program twice a week for 12 weeks [57]. Cytotoxic therapies and its side effects as well as fatigue, muscle weakness and depression may have interfered with the training schedule of the patients leading to the premature withdrawal from the study. Underlining this, our dropped out patients presented significantly higher CRP values and leucocyte counts, less serum albumin, total protein and low hemoglobin concentrations, indicating a higher inflammatory burden, disease severity and advanced cachexia [26]. Importantly, no withdrawal was caused by WB-EMS related discomfort or adverse events emphasizing WB-EMS as a safe training method for this type of patients.

As a hallmark of cachexia, cancer patients often show symptoms of anorexia leading to decreased food intake and thus malnutrition. Both, malnutrition and weight loss have a significant negative impact on the prognosis and outcome of cancer patients and may be prevented by individually adapted nutritional treatment [58–61]. The prevalence of malnutrition among cancer patients is very high and nutritional therapy is often used too late [62, 63]. Therefore, we here monitored nutritional intake to ensure an adequate energy and protein supply and tested the impact of this support also in the control group [25]. At baseline, approximately 60% of WB-EMS and 50% of control patients were at risk for malnutrition (NRS ≥ 3), hinting towards previous weight loss and/or decreased food intake. Within our study, a similar rate of 67.4% of the recruited WB-EMS and 69.0% of the control patients achieved the target protein intake of > 1.0 g/kg/d. Three-quarter of our study patients were able to reach a caloric intake of 25 to 30 kcal/kg/d and higher, whereby approximately one-third had to be supported by additional nutritional supplementation due to decreased food intake linked to cachexia and/or gastrointestinal disorders. However, groups did neither significantly differ in daily nutrient and energy intake nor in the percentage of additionally supplemented patients, suggesting an approximately equal effectiveness of dietary counseling in both groups. Thus, significant differences in muscle mass and body weight at study end may not have been caused by unbalanced nutrient intake. Compared to a recent trial where approximately 50% of the cancer patients achieved the dietary goal of 30 kcal/kg/d and 1.2 g protein/kg/d, the efficacy of our nutritional counseling seems to be satisfactory [64]. Studies suggested that an energy- and protein-rich diet can trigger an increase in body weight and fat mass, but not in lean body mass, especially in advanced cancer patients [2, 46] . Our results support these findings. Although a trend towards increased body weight and muscle mass was seen during the first 4 weeks in the controls, muscle mass progressively declined until week 12 while fat mass percentage was relatively well preserved. The observed significant effect on muscle mass and body weight may be thus mainly caused by the anabolic stimulus of the exercise intervention attenuating anabolic resistance [65].

Further, we evaluated the effect on physical function. Hand grip strength increased in the WB-EMS, but also in the control patients. Hand grip strength has been shown to be independently associated with prognosis and functional characteristics of advanced cancer patients [35] and was also identified as a marker of the nutritional status [66]. About 50% of our study population was prone to be or already malnourished, so an increase in hand grip strength in both groups under nutritional support is not surprising. However, we could not detect significantly higher hand grip strength in the WB-EMS patients. A larger sample size might confirm a superior effect of WB-EMS on grip strength. For determination of the effect on the force of trained muscles we wanted to assess lower limb, abdomen and back muscle strength by isokinetic and isometric strength tests using force plates. However, many study patients were unable to perform this test due to the presence of post-surgical abdominal hernia, osteoporosis or bone metastasis. Lower limb strength correlates with physical function, so we assessed changes in muscle function by the maximum walking distance via the six-minute walk test [36, 67]. Importantly, a significantly longer six-minute walking distance was noted in the WB-EMS compared to the control group, pointing out the high usefulness of WB-EMS in ameliorating the whole muscular status and functional capacity.

Both of our study groups showed marginally better quality of life parameters at study end. The nutritional status is reported to be an important predictor for the quality of life of cancer patients [68]. This may explain the significant improvements in quality of life and fatigue seen within the usual care group. Patients of the WB-EMS group improved significantly in emotional functioning while patients of usual care did not. This observation is consistent with other studies showing a positive impact of exercise on the psycho-social status of cancer patients [69–71]. We think that the positive effects on the trained patients can be attributed to a better handling of everyday life tasks due to significantly enhanced physical function and endurance, both documented here by an increased walking distance and ameliorated performance status (Karnofsky Index).

Physical exercise, in particular strenuous resistance training and also WB-EMS can damage muscle fibres displayed by the release of muscle metabolites such as CK and myoglobin [40, 41]. As those blood markers can effect renal functioning, we monitored the effect of WB-EMS on muscle damage in a subgroup of our patients over the study period. Fortunately, we only observed a moderate increase in muscle enzymes without significant restrictions in renal functioning emphasizing again that supervised WB-EMS training is a safe exercise therapy.

We used BIA instead of the gold standard method of Dual-energy X-ray absorptiometry (DXA) to assess the body composition in our study patients. BIA was demonstrated to correlate with the results obtained from DXA and magnetic resonance tomography [34, 72, 73]. Investigations estimating the reliability of measured body composition parameters by BIA and the wide application range of this technology, also in cancer patients, warranted the use within our study [50, 74, 75]. A huge advantage of the seca instrument that we used in this study is that BIA can be performed easily and very quickly by clinical staff, without distressing patients by elaborated and time-consuming measurement techniques or exposing them to X-rays [76]. We assessed body composition four times in 12 weeks and with DXA, the patients would have been exposed to a very high frequency of radiation. Thus, BIA may be the more innovative and practicable method for the clinical practice and integrative cancer patient care.

Our study has some limitations that may have affected outcomes. The main point of criticism may be the non-randomization of the study patients and the unequal group size that can lead to detected and undetected differences between the two study groups even though patients’ characteristics were well balanced at baseline. This may have increased the chance to detect outcome differences between the study groups. The allocation procedure and the fact that assessors were not blinded may have induced bias in outcome assessment. Also, the possibility that more motivated individuals were included in the exercise group rather than in the control group should be considered here. Patients were allocated with regard to their ability to attend exercise training twice a week. However, individual motivation as potential bias cannot be completely ruled out as a higher level of enthusiasm might have influenced the personal assessment to reconcile a long journey way and the regular attendance at the WB-EMS training. However, this study was conducted as a pilot trial with an explorative character. A future randomized controlled trial accounting for these limitations is needed to confirm our promising results.

## Conclusion

In conclusion, these preliminary results of our pilot study provide evidence that physical exercise in form of WB-EMS is safe and may be an effective exercise technique for advanced-stage cancer patients undergoing treatment. Our combined therapeutical intervention of WB-EMS and nutritional support demonstrates promising effects on muscle maintenance and functioning, and it will be of great interest to further examine this impact of WB-EMS in future larger-scaled randomized controlled trials with additional focus on disease progression and survival.

## Abbreviations

ALT: Alanine transaminase
AST: Aspartate transaminase
BIA: Bioelectrical impedance analysis
CK: Creatine kinase
CRP: C-reactive protein
CUP: Cancer of unknown primary
DXA: Dual-energy X-ray absorptiometry
ECW: Extracellular water
EORTC QLQ-C30: European Organisation for Research and Treatment of Cancer Quality of Life Questionnaire - C30
FACIT: Functional Assessment of Chronic Illness Therapy
FFMI: Fat-free mass index
FM: Fat mass
GFR: Glomerular filtration rate
ICW: Intracellular water
LDH: Lactate dehydrogenase
LMM: Linear mixed models
Mb: Myoglobin
MDRD: Modification of Diet in Renal Disease
NRS 2002: Nutritional risk screening 2002
PhA: Phase angle
SMM: Skeletal muscle mass
SMWT: Six-minute walk test
UICC: Union internationale contre le cancer
WB-EMS: Whole-body electromyostimulation
